# Are They Ready? Trials, Tribulations, and Professional Skills Vital for New Veterinary Graduate Success

**DOI:** 10.3389/fvets.2021.785844

**Published:** 2021-12-23

**Authors:** Addie R. Reinhard, Kristina D. Hains, Bryan J. Hains, Elizabeth B. Strand

**Affiliations:** ^1^College of Agriculture, Food and Environment, University of Kentucky, Lexington, KY, United States; ^2^College of Veterinary Medicine and College of Social Work, University of Tennessee, Knoxville, Knoxville, TN, United States

**Keywords:** veterinary clinical communication, competencies, well-being, veterinary curriculum, veterinary graduates, professional skills, transition to practice

## Abstract

Stress and burnout levels are high among young veterinarians with the transition to practice being particularly challenging. This qualitative study aimed to capture and document the new graduate veterinary experience within the United States and which professional skills are most important for success during the transition to practice. The researchers sought to better understand this challenging transition period and provide insight for veterinary educators who are tasked with preparing new veterinary graduates for day 1 practice readiness. To gain a deeper understanding of the new graduate experience, a focus group was conducted with six veterinarians who recently graduated from four different U.S. veterinary colleges. Several themes arose regarding their experiences in the transition to practice including setbacks and adaptations, self-sufficiency and self-doubt, changing clientele and ethical dilemmas, leadership and conflict, and good vs. bad mentorship. Self-care, conflict management, and client communication were perceived as the most important professional skills for success in the transition to practice. New graduate veterinarians reported that they were least prepared for working with clients with financial constraints and managing conflict. Drawing from this qualitative data, the researchers propose several topics that could be incorporated into professional skills curriculum to further enhance day 1 preparedness of new veterinary graduates to promote well-being in the transition to practice.

## Introduction

Stress levels of veterinarians have been shown to be higher than stress levels within the general population, and among all veterinarians, young veterinarians exhibit the highest stress levels (1, 2). In addition, serious psychological distress was more common among veterinarians who had been in practice for <5 years compared to those who had been in practice longer (3). Many factors contribute to these high stress levels including client complaints and expectations, ethical dilemmas, client financial limitations affecting patient care, educational debt, and professional demands (3, 4). Also, growing evidence suggests that well-being in the veterinary profession may be lowest for young veterinarians (5, 6), and the transition to practice may be a particularly stressful time with a high risk for medical errors and lack of support (7). Training practice-ready veterinarians to be able to cope with these stressors is vital for improving overall mental health among young veterinarians.

The most important skills for success in the transition to practice were found to be professional skills such as communication, confidence and self-efficacy, problem-solving, teamwork, recognizing one's own limits, and decision-making skills (8, 9). Some of these professional skills were ranked more important for early-career success by veterinarians and veterinary students than technical skills (10). Veterinarians must be able to successfully communicate with clients, staff, and colleagues, and self-care skills may be vital for coping with the high levels of stress in the veterinary practice.

Historically, non-technical skills received limited focus in the veterinary medical curriculum, and many veterinarians reported that they had not received any formal training in conflict resolution or self-care during their veterinary training (11). A lack of substantial emphasis on these skills may have resulted from time barriers with a substantial amount of information to cover in the veterinary medical curriculum with little time for extensive training in these non-technical competencies. In addition, it has been suggested that historically, it was thought that veterinary students would learn communication skills and other non-technical skills passively through their clinical year experiences (12).

As veterinary schools are beginning to recognize the importance of these non-technical professional competencies, training for these skills is becoming more widespread (12–15). Recently, the American Association of Veterinary Medical Colleges published a competencies-based framework for veterinary colleges incorporating the competency of “attends to well-being of self and others” [(16), p. 13]. As training in professional skills and well-being is becoming increasingly prevalent within veterinary colleges, questions then arise regarding which specific skills and knowledge should receive focus in these courses.

Gathering a detailed perspective of the experiences of new graduates could provide unique insight into what skills and knowledge should receive focus for veterinary school professional skills courses. This qualitative study aimed to gather rich and detailed data on the new graduate veterinary experience including what professional skills are most important for success during the transition to practice. The primary research questions included:

- What are new veterinary graduates' perceptions of their overall experience transitioning from veterinary student to practicing veterinarian?
- What non-technical skills do recent veterinary graduates feel are most important to success in practice?

## Methods

### Participants and Recruitment

Recent U.S. veterinary graduates were recruited by posting an advertisement in a large veterinary Facebook group with over 20 thousand members. Permission was granted to recruit using this platform from one of the moderators of this group. This advertisement also stated that participants could share the recruitment notice with any individuals fitting the inclusion criteria (U.S. veterinary graduate from the class of 2018 or 2019). Homogeneity and snowball strategies, which are commonly utilized for identifying focus group participants, were used for purposeful sampling to “describe a particular subgroup in-depth” [(17), p. 17]. Six recent veterinary graduates from four different U.S. veterinary colleges from the classes of 2018 and 2019 self-selected to participate in the focus group. No males self-selected to participate in the study. All six recent veterinary graduates were female and worked in private practice. Four were small animal general practice veterinarians, one was a small animal emergency veterinarian, and one was a large animal veterinarian. Pseudonyms will be used in place of real names to protect participant identity (see Table 1).

**Table 1:** Participant information.

**Pseudonym**	**Gender**	**Graduation year**	**Practice type**
Sherry	F	2018	Small animal general practice
Holly	F	2018	Small animal general practice
Jenny	F	2019	Large animal
Jessica	F	2019	Small animal general practice
Olivia	F	2019	Small animal general practice
Sarah	F	2019	Small animal emergency

### Research Design

Focus group methodology was chosen for this study. The 90-min focus group was conducted in January 2020 using a virtual meeting platform. Using a semi-structured interview protocol, participants were asked a series of open-ended questions (Supplementary Table 1) regarding their experiences transitioning into veterinary practice and important professional skills for success in the transition to practice. Participants were able to respond to each other's comments to add additional insight. This group dynamic helps generate deeper dialogue by creating an environment that enables “people to explore and clarify their points of view” [(18), p. 5].

The open-ended interview questions and prompts were collaboratively developed by the researchers to best answer the research questions. Examples of these questions and prompts included, “Describe your experience transitioning from veterinary student to practicing veterinarian” and “What non-clinical skills or knowledge do you feel are most important as a new graduate veterinarian?” IRB approval (IRB #54904) was obtained from the University of Kentucky for conducting this project, and written informed consent was obtained electronically from all participants.

### Data Analysis

Audio from the virtual meeting was recorded and transcribed. The audio transcription was then coded using first cycle eclectic coding which uses an intentional combination of first cycle coding methods that best fit the data. Codes from qualitative data are “essence-capturing and essential elements of the research story that, when clustered together according to similarity and regularity (i.e., a pattern), actively facilitate the development of categories and thus analysis of their connections” [(19), p. 9]. For this study, *in vivo*, process, and descriptive coding were used in first cycle coding. For second cycle coding, frequent and significant codes were then categorized based on similarity using focused coding. Code weaving was used to create a final narrative to give a detailed portrayal of the new graduate experience.

### Limitations

Due to the small number of participants in this study, it may be difficult to generalize or make assumptions about the general population of new veterinary graduates. In addition, all participants in this study were female and represented graduates from only four U.S. veterinary colleges limiting the generalizability of the data. However, the purpose of qualitative research is not to provide statistically significant results, but rather, to provide rich and detailed data to better understand a particular experience or phenomenon. For future studies, a larger population of new graduates may be beneficial to make generalizations. Finally, participants self-selected to be in the focus group study which may introduce self-selection bias into the data.

## Results

Regarding the experiences during the transition to practice, five themes arose and were grouped into the category *Trials and Tribulations of New Veterinary Graduates* including setbacks and adaptation; self-sufficiency and self-doubt; changing clientele and ethical dilemmas; leadership and conflict; and good vs. bad mentorship. Three themes were grouped within the category of *Skills Vital for Success in the Transition to Practice* including client communication, conflict management, and self-care.

### Trials and Tribulations of New Graduate Veterinarians

… *first year out was the hardest. Learning to, you know, stand on my own two feet and not have an army of people to be able to ask questions to. (Sherry)*

#### Setbacks and Adaptation

The start of the veterinary career was sometimes associated with frustration, setbacks, adaptation, and even psychological ramifications. Two veterinarians mentioned issues with sleeping early in their careers. The sudden increase in caseload was a particular area of stress for a few veterinarians. In addition, both of the 2018 graduates, Sherry and Holly, mentioned that they had already changed practices once within the first 18 months of their careers.

Three of the veterinarians mentioned experiencing discrimination based on their age, ethnicity, and gender. Jenny spoke candidly about her experience as a new graduate large animal veterinarian sharing, “And then I think another frustrating thing for me is being a woman working in large animal. The sexist comments that get made. I get very, very frustrated with those.” Olivia had also experienced discrimination early in her career stating,

I have had some setbacks. There was some people that look at me and say, “Are you the tech or are you the assistant, where's the doctor?” And I'm like, “No, I'm here, I'm your doctor.” And then other people flat out telling me, “I don't want to interact with you, can you please get me the other doctor. I don't feel comfortable talking to *your people*.”

When discussing euthanasia, a few of the vets mentioned having difficulty coping particularly when the case was associated with an ethical dilemma. Euthanasia was mentioned as emotionally challenging, and Sarah shared,

Just the sheer number of euthanasias that I do I think was really, is really, still really difficult for me… the first few months the other doctors would sort of tease me a little bit, not in a mean way, but I would always be in tears after coming out of euthanasia and they would always say, “Oh you'll get past that.”

#### Self-Sufficiency and Self-Doubt

As the veterinarians entered private practice, they often transitioned from having an “army of people” to support them to an expectation of self-sufficiency. This self-sufficiency was often accompanied with a sense of self-doubt. Jessica shared:

And then about maybe two months after I started… it suddenly hit me that I was responsible for people's pets, and people were depending on me, and I was terrified of screwing up… I started doubting myself and started second-guessing myself.

Self-doubt and fear of making mistakes were mentioned during the focus group by three of the veterinarians. Sarah, an emergency veterinarian, stated that she was terrified that she “was going to be on the end of a shift and just really tired and draw up the wrong amount of medicine or just screw up.” Holly recounted a particular mistake that she made early in her career that caused lasting remorse.

#### Changing Clientele and Ethical Dilemmas

Veterinary teaching hospitals are typically referral hospitals which leads to an inherently different clientele than that of many private practices. This shift in clientele, particularly when working with clients with low incomes, was mentioned during the focus group. Upon entering private practice, four of the veterinarians in the focus group mentioned client financial limitations as a difficult obstacle faced.

Sometimes it was difficult to cope with not being able to provide the gold standard treatment. Holly seemed conflicted during the “transition from kind of ivory tower best medicine to like what the people can be able to afford which of course you don't want to compromise the medicine that you are practicing because of the clientele that you see.” Some cases discussed during the focus group that were associated with client financial limitations led to ethical dilemmas which contributed to stress at the start of practice. Holly discussed the challenge of navigating ethical dilemmas:

I recommended microchipping the cat and he was like, “I'm not going to microchip the cat if the microchip's just going to end up in coyote poop.” Which shocked me… The struggle that I have is that the cultural… ideas about the role of the animal in the family or the purpose or job of the animal in the family… And I'm a person who thinks animals are part of my family.

#### Leadership and Conflict

New veterinary graduates often take on a leadership role when entering practice where they must delegate tasks to support staff. For some of the recent graduates, this new leadership role came more naturally and was associated with ease in building rapport with clients. For others, taking on this leadership role was not as straightforward. One difficulty three of the veterinarians shared was building trust and rapport with either clients or support staff. Olivia demonstrated this by sharing, “Even you having that leadership role and knowing and trying to keep that within your staff, you even get questioned every single time.”

Conflict with clients and support staff was mentioned frequently. Some of the new graduates described frustration and conflict when an owner or veterinary technician asked them to consider alternatives to their protocols. For example, Holly shared, “So I have a protocol. First, we give pre-meds. We give butorphanol. I don't care if the tech thinks that the animal isn't that painful, that's just how I do it.”

#### Good Mentorship vs. Bad Mentorship

A common theme voiced by almost all the veterinarians in the focus group was mentorship. Most of the new graduates described an overall positive view of their mentorship, and good mentorship seemed to be very important to new graduates. A good mentor was described as someone who was available and willing to answer questions. In addition, a good mentor was described as an advocate—someone supportive and trusting of new graduate's medical knowledge. Finally, a good mentor was described as someone who could empathize with the veterinarian regarding difficulties they were experiencing. Only a few new graduates had negative comments regarding their mentorship. Holly recounted the story of her first few months in practice where her mentor had to go on leave when she first started, and when she returned “my mentor was just not, she was overworked… and not really able to be a good mentor.”

### Skills Vital for Success in the Transition to Practice

#### Client Communication

Client communication was one of the most commonly mentioned professional skills seen as important during the early career. Simplifying medical jargon and building rapport and trust among clients were seen as important communication skills among new veterinary graduates. “Advocating for best medicine” during client communication was seen as an important communication skill among three of the veterinarians, and several veterinarians expressed that empathy was an important skill, yet they were often unsure of how to proceed with a case in the context of limited owner financial resources.

#### Conflict Management

Conflict management was seen as an important skill for new veterinary graduates, but many of the new graduates did not feel prepared or confident in navigating conflict. Several of the veterinarians mentioned that conflict management was one of their least confident skills. Two of the veterinarians mentioned that they were unsure how to handle conflicts when two pet owners disagreed on the course of action for the pet. One veterinarian mentioned that her school had prepared her well on how to handle client conflicts but not conflict within the staff.

#### Self-Care

All veterinarians in the focus group mentioned some form of self-care was important during the transition to practice. Three of the veterinarians mentioned that it was important to understand your limits and when you needed to “ask for help.” Several of the veterinarians discussed the importance of social support from peers and veterinary social media groups during the transition to practice. Self-care was seen as an important skill, but several of the veterinarians mentioned that they had not received much training in self-care, burnout, or mental health issues. While many of the veterinarians shared that self-care training was important, Sarah mentioned that no level of preparedness would be sufficient sharing, “No matter how hard they try to prepare you… some of the stuff you are just going to have to figure out how to deal with when you're there.”

## Discussion

This qualitative study provided detailed data on new veterinary graduates' perceptions of their experiences in the transition to practice. By analyzing the experiences and challenges of the focus group participants, topics are suggested for incorporation into professional skills training to enhance day 1 preparedness of new veterinary graduates (see Table 2). This list of topics is not meant to be all-inclusive, but rather, draws from the experiences of the new graduates within this study to offer suggestions as to what could be potentially incorporated into professional skills training.

**Table 2:** New graduate focus group themes and related professional skills topics.

**Category**	**Theme**	**Professional skills curriculum topic**
Trials and tribulations of new graduate veterinarians	Setbacks and adaptation	Responding to discrimination Basic self-care Euthanasia and grief
	Self-sufficiency and self-doubt	Disclosing medical errors Coping with making a mistake
	Changing clientele and ethical dilemmas	Spectrum of care, incremental care Ethical decision-making Coping with moral distress
	Leadership and conflict	Leadership Building trust and rapport Professional identity development
	Good vs. bad mentorship	Seeking quality mentorship
Skills vital for success in the transition to practice	Client communication	Communicating with clients with low incomes
	Conflict management	Navigating conflict with support staff Managing conflict between clients
	Self-care	Burnout and compassion fatigue Techniques to cope with stress Encouraging help-seeking Personal identity development

Several challenges were identified in the focus group that may contribute to high levels of stress and relatively low levels of well-being among new veterinary graduates (1, 6). The start of the veterinary career was associated with a sudden expectation of self-sufficiency and responsibility that was coupled with a sense of self-doubt. Self-doubt and fear of making mistakes were mentioned several times during the focus group, and this finding was consistent with a previous study that found three out of four veterinarians had made a mistake within the first 18 months of their career, and these mistakes often had an emotional impact (7). Some of the other challenges new veterinary graduates reported included discrimination based on age, ethnicity, and gender, sleepless nights, and adapting to overwhelming caseloads. Euthanasia was also considered emotionally challenging for some especially when it involved an ethical dilemma. It is estimated that U.S. veterinary schools offer an average of around 7 h of training in death, dying, and bereavement (20). When considering professional skills training to support veterinary well-being during the transition to practice, special attention should be given to self-care, responding to discrimination, mistake-making, and training on grief and bereavement related to euthanasia including coping with one's own grief.

Client financial limitations are extremely common with ~1 in every four households experiencing a barrier to receiving veterinary care (21). One of the most common ethical dilemmas faced in veterinary practice was financial barriers to pet care (22), and over half of veterinarians experienced moderate to very high stress when faced with financial limitations affecting pet care (4). During the focus group, four of the new graduates shared that a challenge faced in the transition to practice was client financial limitations to care. Some participants stated that they were unsure of how to proceed if a client declined their initial recommendations due to financial limitations. This is consistent with previous findings that suggested one of the communications skills that veterinary students were least confident in was discussing financial issues with clients (23).

While teaching veterinary students how to provide gold-standard patient care is paramount, an additional focus should be placed on learning how to provide care over a spectrum as “acceptable outcomes can be achieved at many points along the spectrum of care” [(24), p. 1389]. In addition, training could be provided to veterinary students on how to cope with moral distress which is considered severe emotional distress that may be perpetuated by morally challenging stressors (25). Objective Structured Clinical Examination (OSCE) techniques have been used to evaluate knowledge in navigating ethical dilemmas in both human and veterinary medicine (26, 27) and could be an effective evaluation tool for ethical cases incorporated into the curriculum.

Taking on a leadership role and the subsequent conflict associated with that role arose as a common theme during the focus group. New graduates act as a leader in the care of the animal which often requires communication and collaboration with clients and support staff to provide care. Some focus group participants described conflict and frustration arising when clients or support staff asked them to deviate from gold standard protocols. These statements were consistent with a diagnosis-focused identity which was described by Armitage-Chan & May as placing “greatest value on patient diagnosis and treatment” [(28), p. 6]. To better prepare students for navigating this type of conflict, it has been recommended that a stronger focus be placed on professional identity development throughout the veterinary curriculum (29). In addition, it has been suggested that identity development and increasing self-awareness are major aspects of leadership development (30).

Mentorship was mentioned frequently during the focus group. The new veterinary graduates shared that a good mentor is willing to answer questions, advocates and supports them, and empathizes with them regarding their challenges. A previous study on mentorship found that veterinary mentors often have personality traits of extroversion, conscientiousness, and openness (31). Openness of mentors to answer questions and seek new graduate's feedback seemed to be especially important to the new graduates in this study. It is recommended to teach students how to find good mentorship during the transition to practice as good mentorship was perceived to be highly important to new graduates. In addition, finding a good mentor may be particularly important as new veterinary graduates who reported having inadequate support in the transition to practice had higher levels of stress (32). A few of the focus group participants who mentioned that they had good mentors still experienced challenges in the transition to practice which demonstrates a need to assess structures and training that could further improve mentorship. Future research could evaluate what makes a good or bad mentor as well as establish standards for mentorship.

Three themes arose when new veterinary graduates were asked about the most important non-technical skills for success in the transition to practice including client communication, conflict management, and self-care. In previous studies, client communication was ranked as one of the most important skills or attributes at the start of the veterinary career (8, 33), and in one study, 88% of recent veterinary graduates stated that client communication was very important (9). Conflict management was perceived as a vital skill for success in the transition to practice, yet, for a few focus group participants, this was also one of their least confident skills. This finding does not come as a surprise as 71% of North American veterinarians reported that they did not receive training in conflict resolution (11).

Self-care was seen as one of the most important skills for success during the transition to practice. The finding that new graduates were placing a strong emphasis on self-care was encouraging as the Merck Animal Health Veterinary Well-being Study suggested that well-being was lowest for young veterinary graduates (5). Researchers from Colorado State University suggested providing additional training in well-being during the veterinary curriculum (34). Increased training in topics surrounding self-care may be important in preparing new graduates for coping with the stressors of clinical practice.

## Conclusion

One main implication of this research is to recognize the important data that can be gained by seeking the story and listening to the voices of new veterinary graduates. Focus groups could be conducted with new veterinary graduates from a veterinary school to evaluate areas of deficiency and gaps in the curriculum by identifying challenges that the new graduates are facing. It should also be noted that just because a competency is perceived to be of high importance to new veterinary graduates, it does not necessarily indicate that a competency improves day 1 practice readiness (35). The value comes from uncovering the unique challenges faced by new graduates to identify areas veterinary educators could better prepare their graduates in order to promote well-being during the transition to practice.

Veterinary educators should make every effort to best prepare their graduates for practice readiness but should also consider that no level of training may be sufficient at fully preparing new graduates for the challenges of practice. This also suggests that it may be beneficial to offer professional skills training at the start of the veterinary career when many of these challenges are first being encountered which could be done through professional development programs, leadership training, continuing education, or formal mentorship programs.

Finally, as some non-technical professional skills were ranked more important than technical skills in the success in the transition to practice (10), veterinary educators should place a high emphasis on teaching these skills within the veterinary curriculum. This study and others that have evaluated professional skills necessary for success in the transition to practice could be used as a foundation to teach professional skills which may lead to increased new graduate preparedness. Increased new graduate confidence in professional skills may potentially decrease stress in the transition to practice and may result in higher quality patient care.

## Data Availability Statement

The datasets presented in this article are not readily available because data contain potentially identifying information. Requests to access the datasets should be directed to addiereinhard@gmail.com.

## Ethics Statement

The studies involving human participants were reviewed and approved by University of Kentucky Institutional Review Board (IRB #54904). The patients/participants provided their written informed consent to participate in this study.

## Author Contributions

AR, ES, and KH conceived of the study and formulated focus group questions. AR conduced the focus group and drafted the initial version of the manuscript. AR analyzed and coded transcript with the assistance of BH and ES. All authors edited the manuscript and approved the final version.

## Conflict of Interest

The authors declare that the research was conducted in the absence of any commercial or financial relationships that could be construed as a potential conflict of interest.

## Publisher's Note

All claims expressed in this article are solely those of the authors and do not necessarily represent those of their affiliated organizations, or those of the publisher, the editors and the reviewers. Any product that may be evaluated in this article, or claim that may be made by its manufacturer, is not guaranteed or endorsed by the publisher.
