# Supplementary material for: Are They Ready? Trials, Tribulations, and Professional Skills Vital for New Veterinary Graduate Success
Source: Front Vet Sci. 2021 Dec 23;8:785844. doi: 10.3389/fvets.2021.785844 (PMC8732754; doi:10.3389/fvets.2021.785844)
Supplement: Supplementary file 1 [file Table_1.DOCX]

| **Supplementary Table 1. New Graduate Focus Group Questions** | |
| --- | --- |
| **Question Type** | **Question** |
| Introductory Question | 1. Please share with us the type of veterinary practice you are working in and how long you have worked at that practice. |
| Key Questions | 2. Describe your experience transitioning from veterinary student to practicing veterinarian.  3. What aspects of the transition from veterinary student to practicing veterinarian did you not feel prepared for?  4. What types of resources would have been helpful to you during your transition to practice?  5. What non-clinical skills or knowledge do you feel are most important as a new graduate veterinarian?  6. What non-clinical skills did you feel least confident in during the transition to practice?  7. If you had to go through the transition from student to practicing veterinarian again, what non-clinical skills or knowledge would you have liked to learn more about during the first six months of practice?  8. Describe the level of connection that you currently feel in the veterinary community. |
| Closing Questions | 9. What advice would you give to veterinary students transitioning into practice? |
